# Reliability of measurements on lateral ankle radiographs

**DOI:** 10.1186/s12891-016-1150-4

**Published:** 2016-07-18

**Authors:** Changjun Guo, Yuan Zhu, Mu Hu, Lianfu Deng, Xiangyang Xu

**Affiliations:** Department of Orthopaedics, Rui Jin Hospital North, Shanghai Jiao Tong University School of Medicine, Shanghai, China; Department of Orthopaedics, Rui Jin Hospital, Shanghai Jiao Tong University School of Medicine, Shanghai, China; Shanghai Key Laboratory for Prevention and Treatment of Bone and Joint Diseases with Integrated Chinese-Western Medicine, Shanghai Institute of Traumatology and Orthopaedics, Rui Jin Hospital, Shanghai Jiao Tong University School of Medicine, Shanghai, China

**Keywords:** Ankle, Lateral radiographs, Interobserver study, Reliability

## Abstract

**Background:**

The aims of our study were to evaluate the validation of measurement of weight-bearing lateral radiographs. Two hypotheses were tested: the measurements on the lateral radiographs are reliable, and a theoretical limit could be identified when a surgeon can “eyeball” an incongruous ankle joint on lateral radiographs.

**Methods:**

To test the first hypothesis, 3 experienced ankle surgeons evaluated 50 normal weight-bearing lateral radiographs of patients. The measurements assessed were the tibial lateral surface angle (TLS), the distance from the center of the talar joint circle to the longitudinal axis of the tibia (x) and the displacement from the center of the talar articular joint circle to the center of the distal tibia articular joint circle (d). To test the second hypothesis, we used CAD software to create schematic diagrams on which lateral radiographs of the ankle joint were not parallel (*d* = 1, 2, 3, 4 mm). Five experienced ankle surgeons were asked to judge whether the ankle articular surfaces were parallel. Intraobserver reliability was determined using the intraclass correlation coefficients (ICCs) and interobserver agreement by the Kendall coefficient of concordance.

**Results:**

First, the intraobserver reliability was high (Cronbach’s alpha >0.80) with regard to radiographic measurements according to the ICC. Significant interobserver disagreement was found (Kendall tauB, *p* < 0.01) using the Kendall concordance coefficient. Second, when the *d*-value was 4 mm, all the observers identified the incongruous ankle joint at two separate times.

**Conclusions:**

Consultation with experienced foot and ankle surgeons and precise definitions for lateral measurement assessments do not guarantee a high level of agreement. Surgeons can observe an incongruous ankle joint on lateral radiographs when the *d*-value is 4 mm.

## Background

The ankle sustains the weight of the body, and when a fracture occurs, restoration of the anatomical alignment during treatment is of foremost importance. It is generally agreed that poor ankle articular reduction results in accelerated development of posttraumatic ankle osteoarthritis [1, 2].

Radiographs of the ankle provide information regarding the integrity of the ankle mortise. Numerous linear and angular relationships have been described in the literature with respect to the anteroposterior (AP) and mortise radiograph characteristics of the ankle joint that are used to evaluate anatomic alignment [3–6]. Many criteria have been documented, including a medial clear space (MCS) less than 4 mm and greater than 1 mm, a tibia fibula clear space (CS) less than 5 mm, and tibia fibula overlap (OL) less than 10 mm [7, 8].

In our clinical cases of postoperative ankle fracture in which AP and mortise radiographs show relatively good realignment, some patients still have residual complaints and poor clinical results. In such cases, we have reviewed the roentgenograms and found different deviations of alignment in lateral radiographs. However, there has not been a report on validation of measurements of the lateral view of ankle alignment.

**Fig. 1:**
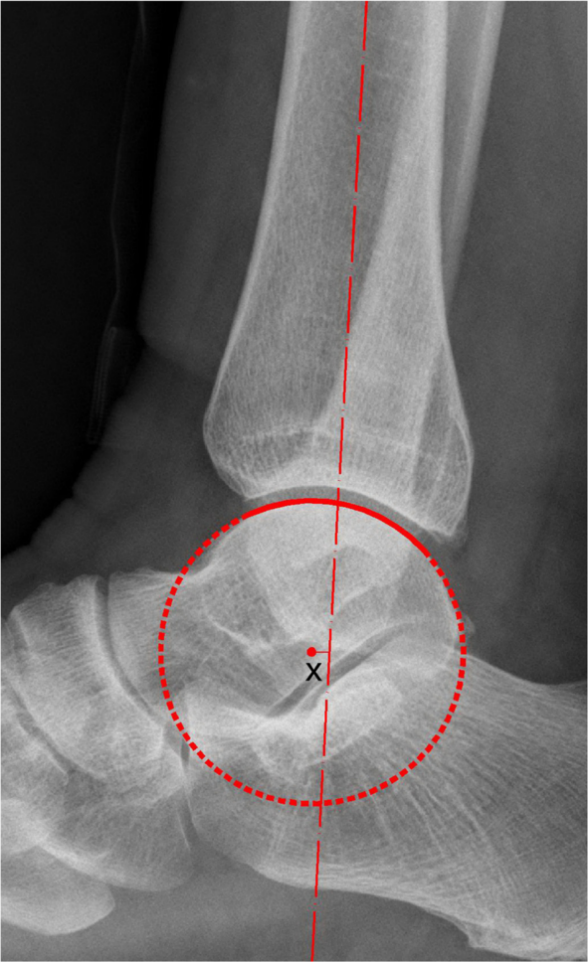
The distance (x) from the center of the talar joint circle to the longitudinal axis of the tibia

**Fig. 2:**
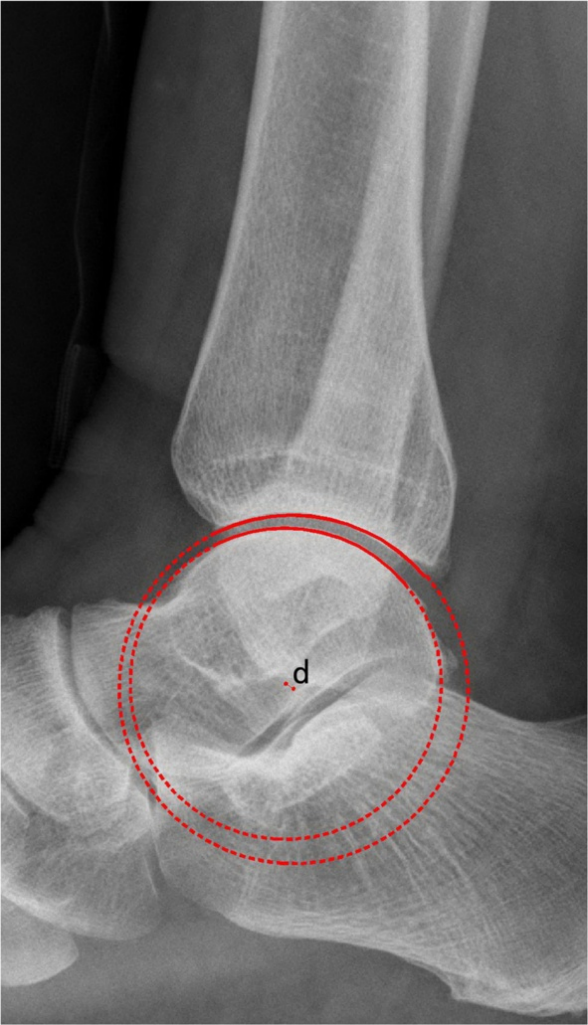
The displacement (d) from the center of the talar articular joint circle to the center of the distal tibia articular joint circle

On a lateral radiograph, the tibiotalar articular surfaces should be parallel, with no extrusion of the talus out of the mortise [9]. The distance from the center of the talar joint circle to the longitudinal axis of the tibia (Fig. 1), and x is considered when evaluating the position of the talus in the ankle joint [10]. In the current study, we investigated the displacement from the center of the talar articular joint circle to the center of the distal tibia articular joint circle (Fig. 2) in lateral radiographs, and d was used to visually assess the parallelism of the articular surface.

The aims of our study were to evaluate the validation of measurement of weight-bearing lateral radiographs. We collected precise measurements from weight-bearing lateral radiographs of the ankle and hypothesized that measurements on the lateral radiographs are reproducible and reliable. A theoretical limit could be identified when a surgeon can “eyeball” an incongruous ankle joint on lateral radiographs using the d measurement.

## Methods

### Patients

Our study included 80 consecutive patients at our department who had symptoms of chronic pain, functional damage, or post-traumatic ankle arthritis. All the patients had both ankle weight-bearing plain radiographs due to the unilateral lesions of the ankle. The weight-bearing plain radiographs of the ankle were set up for the first patient according to the predefined protocol. The predetermined quality criteria were the same as in previous studies: the medial joint line of the talus had to superimpose on the lateral joint line, and the distal fibula had to project onto the posterior third of the distal tibia [10]. According to the criteria, 30 patients were excluded because of the subtalar position (10 patients) and oblique lateral radiographs (20 patients). The remaining 50 patients (23 men and 27 women) with a mean age of 45 years (range 18–72) were included in our study.

### Radiographic measurements

**Fig. 3:**
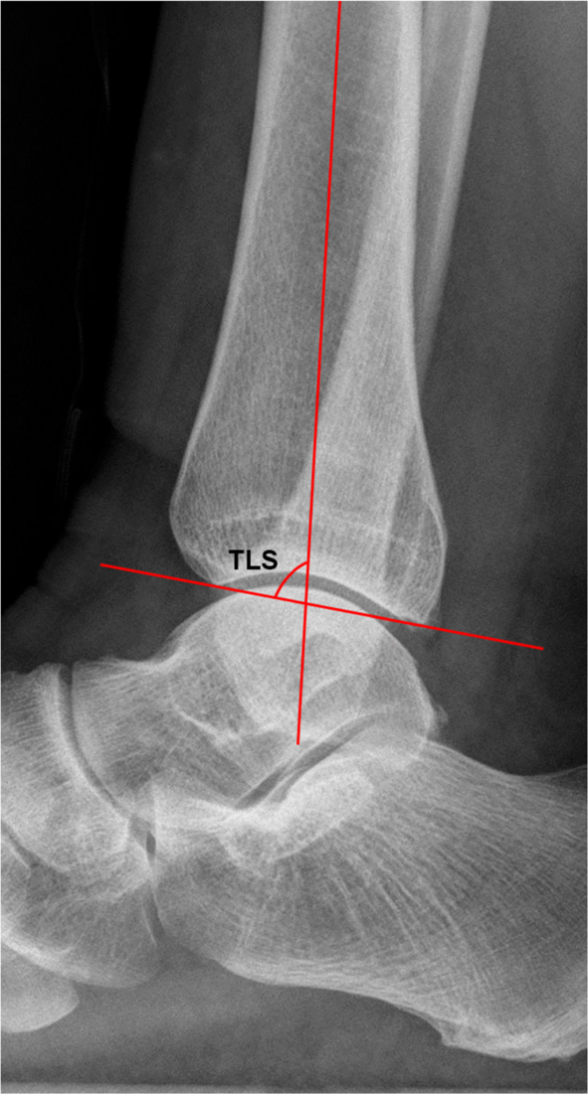
The images for tibial lateral surface angle (TLS)

To test the first hypothesis, 50 normal lateral radiographs were digitally collected through the Picture Archiving Communication System (PACS, EBM Technologies Incorporated, Taiwan, China) between July 2015 and January 2016. We first set the scale distance through the PACS system, and then transferred the images to the CAD (computer-aided design, Autodesk Company, California, USA) software for precise measurement. The tibial lateral surface angle (TLS) was measured (Fig. 3). TLS is an angle between the tibial axis and the distal tibia articular surface that is drawn between the anterior and posterior margins of the tibial plafond [11]. We manually traced a sector of a circle to the talar joint and the distal tibia articular joint. The x was defined as the distance from the center of the talar joint circle to the longitudinal axis of the tibia [10]. The d was defined as the displacement of the center of these two different circles.

Before the start of the analysis, five normal weight-bearing lateral radiographs were evaluated together to ensure that the observers drew angles in the same manner. Three experienced foot and ankle surgeons completed our study. For intraobserver reliability, the same radiographs were measured by each observer after two weeks.

**Fig. 4:**
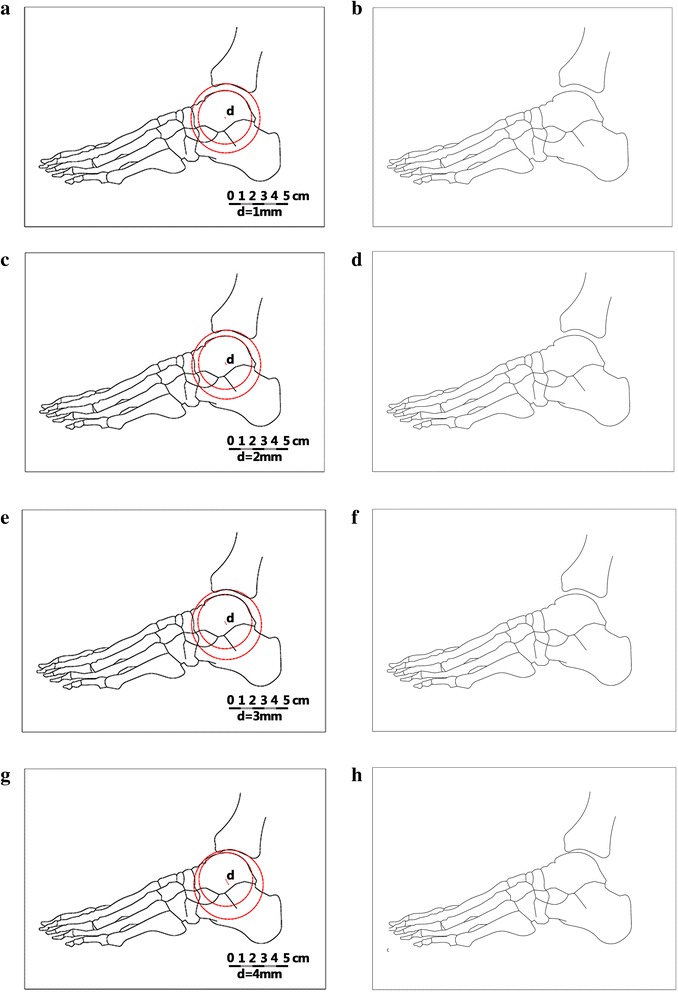
The schematic diagrams on which lateral radiographs of the ankle joint were not parallel. **a**, **c**, **e**, **g**: *d*-value is marked; **b**, **d**, **f**, **h**: *d*-value is not marked. **a** and **b** (*d* = 1 mm); **c** and **d** (*d* = 2 mm); **e** and **f** (*d* = 3 mm); **g** and **h** (*d* = 4 mm)

To test the second hypothesis, we used the CAD software to create schematic diagrams on which lateral radiographs of the ankle joint surfaces were not parallel (*d* = 1, 2, 3, 4 mm; Fig. 4). The diagrams corresponded to the supposed clinical scenario (the talus was extruded anteriorly). Five experienced foot and ankle surgeons, who were blinded to the marked diagrams, were selected and asked to view the slide show and mark “yes” or “no” in response to the question “Are the articular surfaces parallel?”

### Statistical analysis

Intraobserver reliability was determined by the intraclass correlation coefficients (ICCs) for continuous data. A Cronbach’s alpha greater than 0.80 indicated perfect reliability. Interobserver agreement was determined by the Kendall coefficient of concordance (Kendall’s W) when measuring the different parameters for each subject. Kendall’s W varies between 0.0 (no agreement) and 1.0 (maximum agreement). The differences were considered statistically significant if the *p*-value was less than 0.05. All statistical analyses were performed using SPSS version 22.0 software (SPSS Inc., Chicago, IL).

## Results

First, in the standard lateral radiographs, the x, d and TLS were measured by three observers in two separate viewings (Table 1). In the two viewings, the first observer’s measurements were 2.12 ± 0.34 mm and 2.24 ± 0.32 mm for the *d*-value, 2.24 ± 0.43 mm and 2.22 ± 0.54 mm for the *x*-value and 81.9 ± 1.92 and 83.2 ± 1.96 for the TLS-value. The *d*-value (3.20 ± 0.27 mm vs 3.12 ± 0.28 mm), *x*-value (3.13 ± 0.19 mm vs 3.01 ± 0.18 mm) and TLS-value (78.3 ± 3.64 vs 82.2 ± 3.68) by the second observer. The *d*-value (4.23 ± 0.26 mm vs 4.33 ± 0.25 mm), *x*-value (2.07 ± 0.21 mm vs 2.11 ± 0.20 mm) and TLS-value (79.1 ± 3.67 vs 81.1 ± 3.69) by the third observer.

**Table 1:** Lateral radiographic measurement of the 50 selected subjects (‾x ± SD)

	d(mm)		x(mm)		TLS(°)	
	First	Second	First	Second	First	Second
Observer1	2.12 ± 0.34	2.24 ± 0.32	2.24 ± 0.43	2.22 ± 0.54	81.9 ± 1.92	83.2 ± 1.96
Observer2	3.20 ± 0.27	3.12 ± 0.28	3.13 ± 0.19	3.01 ± 0.18	78.3 ± 3.64	82.2 ± 3.68
Observer3	4.23 ± 0.26	4.33 ± 0.25	2.07 ± 0.21	2.11 ± 0.20	79.1 ± 3.67	81.1 ± 3.69

*TLS* tibial lateral surface angle

The intraobserver reliability, as determined by the ICC, was very high with regard to radiographic parameters (Table 2). The intraobserver reliability across three observers yielded an alpha statistic greater than 0.8, indicating perfect consistency in the observers’ evaluation of identical radiographs in two separate viewings. The interobserver agreement between three foot and ankle surgeons was significantly different for the *x*-value, *d*-value and TLS in two separate measurements (Table 3). These results show that the measurements of the weight-bearing lateral radiographs showed incomplete agreement for all radiographs evaluated.

**Table 2:** Intraobserver reliabilities of lateral radiographic measurement for the 50 selected subjects (ICC)

	Observer1 (alpha,95%CI)	Observer2 (alpha,95%CI)	Observer3 (alpha,95%CI)
d	0.81(0.64–0.88)	0.93(0.88–0.96)	0.90(0.83–0.95)
x	0.96(0.94–0.98)	0.91(0.84–0.95)	0.82(0.68–0.89)
TLS	0.95(0.92–0.97)	0.95(0.91–0.97)	0.96(0.94–0.98)

*ICC* intra-class correlation coefficients, *TLS* tibial lateral surface angle

**Table 3:** Interobserver reliabilities of lateral radiographic measurement for the 50 selected subjects

	First time		Second time	
	*X* ^2^	*p*	*X* ^2^	*p*
d	92.6	*p* < 0.001	100	*p* < 0.001
x	72.2	*p* < 0.001	54.9	*p* < 0.001
TLS	30.0	*p* < 0.001	27.1	*p* < 0.001

*TLS* tibial lateral surface angle

Second, five observers evaluated schematic diagrams with different *d*-values in two separate viewings. When the *d*-value was 1 mm or 2 mm, all the observers identified the congruous ankle joint on lateral radiographs in both viewings. When the *d*-value was 3 mm, only two observers identified the incongruous ankle joint, each in one viewing. When the *d*-value was 4 mm, all the observers identified the incongruous ankle joint in both viewings. These results showed that a theoretical *d*-value of 4 mm enabled surgeons to “eyeball” an incongruous ankle joint on lateral radiographs.

## Discussion

Our study showed the outcomes of precise measurements of weight-bearing lateral radiographs, including the *x*-value, *d*-value and TLS. Significant interobserver disagreement was found using the Kendall concordance coefficient. The measurements of the lateral radiographs were not reliable. When the *d*-value was 4 mm, all the observers identified the incongruous ankle joint in two separate viewings.

Malalignment of the anatomical axis of the ankle joint may alter the normal load distribution and articular congruency, resulting in chronic pain, functional damage, and, ultimately, post-traumatic ankle arthritis [12–14]. Therefore, successful restoration of anatomical alignment with full mortise congruency and joint stability is vital to the treatment of ankle fractures.

It is well known to orthopedic surgeons that poor articular reduction leads to accelerated development of posttraumatic ankle osteoarthritis [1]. Pagenstert et al. [15] reported that the mid-axis of the tibia passes through the center of talar joint surface on a standing lateral ankle radiograph, but did not report any measurements. Magerkurth et al. [16] reported the *x*-value of a normal ankle joint.

In our study, we attempted to evaluate the reliability of a precise measurement on lateral radiographs by testing two hypotheses: 1) measurements on lateral radiographs are reliable and 2) a theoretical limit could be identified at which a surgeon can “eyeball” an incongruous ankle joint on lateral radiographs. The first hypothesis was rejected. The intraobserver reliability was greater than 0.8, indicating perfect consistency in the observers’ evaluation of identical radiographs in two separate viewings. However, the measurements for all three values—x, d and TLS—were not reliable. The significance of this finding, which was supported by lack of interobserver reliability, is far-reaching. It questions the validity of using measurements to determine whether an ankle joint is congruent. In one report, the mean *x*-value for a normal ankle joint was 1.7 mm; however, a variation of more than 10 mm may be found [10].

Based on our findings, the second hypothesis was validated. A lateral talar displacement, by even 1 mm, will produce a 42 % reduction in the area of tibiotalar contact, leading to degenerative arthritis [17]. Although the small talar displacement led to early ankle osteoarthritis, all observers identified the congruous ankle joint on lateral radiographs when the *d*-value was 1 mm or 2 mm. It seems clear that a theoretical *d*-value of 4 mm could be identified when a surgeon can “eyeball” an incongruous ankle joint on lateral radiographs. The ability to visually detect a minimum *d*-value of 4 mm is also clinically useful. When surgeons are able to recognize during an operation that the articular surfaces are not parallel, it means that the *d*-value is more than 4 mm. This suggests that surgeons need to reexamine the impact of articular congruity.

One study reported that displaced osteochondral fragments of 5 mm in width were not consistently recognizable on lateral fluoroscopic radiographs [18]. In that study, the 5 mm malreduction was in the sagittal plane of cadaveric specimens, but we first reported the *d*-value in lateral radiographs and used it to assess the parallelism of the articular surfaces in the coronal plane of the schematic diagrams.

In our study, accurate assessment of tibiotalar displacement in the lateral view depended on accurate weight-bearing lateral radiographs. It would be very important to obtain such radiographs intraoperatively or immediately after surgery when this information would be of greatest value in preventing later problems. Therefore, we suggest that the ankle position should be neutral using C-arm monitoring during or immediately after surgery in order to obtain normal lateral radiographs according to the above criteria.

Our study had two limitations. First, this is an artificial scenario to evaluate whether the ankle articular surfaces are parallel. Second, our measurements depended on good radiographic technique. Poor radiographic technique may contribute to diagnostic error by invalidating established measurements.

## Conclusions

We analyzed the outcomes of precise measurements using a weight-bearing lateral view, including the *x*-value, *d*-value and TLS. Input from experienced foot and ankle surgeons could not guarantee a high level of agreement. Surgeons could observe an incongruous ankle joint on lateral radiographs when the *d*-value was 4 mm. However, further investigation is needed to determine the relationship between articular reduction and lateral measurements in the ankle.

## Abbreviations

AP, anteroposterior; CAD, computer aided design; CS, clear space; ICCs, intraclass correlation coefficients; MCS, medial clear space; OL, overlap; PACS, Picture Archiving Communication System; TLS,tibial lateral surface
